# HLA-G high-expressor 3’UTR markers are linked to gastric cancer development and survival

**DOI:** 10.1007/s00262-024-03771-w

**Published:** 2024-11-16

**Authors:** Christian Vaquero-Yuste, Ignacio Juarez, Marta Molina-Alejandre, Elisa María Molanes-López, Alberto Gutiérrez-Calvo, Adela López-García, Inmaculada Lasa, Remedios Gómez, Antonio Arnaiz-Villena, Jose Manuel Martín-Villa

**Affiliations:** 1 Departamento de Inmunología, Oftalmología y ORL, Facultad de Medicina, Universidad Complutense de Madrid, Madrid, Spain; 2 Departamento de Estadística e Investigación Operativa, Facultad de Medicina, Universidad Complutense de Madrid, Madrid, Spain; 3 Servicio de Cirugía General y Aparato Digestivo, Hospital Universitario Príncipe de Asturias, Madrid, Spain; 4 Instituto de Investigación Sanitaria Gregorio Marañón, Madrid, Spain

**Keywords:** HLA-G antigens, Stomach neoplasm, Prognosis, Haplotypes, Clinical relevance, Tumor escape

## Abstract

Gastric cancer ranks fifth in both world prevalence and lethality, with a 5-year survival of less than 30%. HLA-G, a non-classical class I HLA gene, has emerged as a potential marker for cancer susceptibility and prognosis due to its immunomodulatory properties. Its level of expression is regulated by polymorphisms in the 3’ untranslated region (3’UTR) polymorphisms, which form various combined haplotypes (UTR-1 to -9). In this study, we examined HLA-G 3’UTR polymorphisms in paired tissue samples from 111 patients with gastric adenocarcinoma and 119 healthy controls. Polymorphism analysis was performed using PCR and Sanger sequencing, followed by statistical analysis using SNPStats software. Survival analysis was conducted using Kaplan–Meier curves and multivariate Cox regression models. High-expressor HLA-G 3’UTR haplotypes (UTR-1 and UTR-6) were significantly associated with gastric cancer susceptibility, indicating a potential role in tumor immune evasion. Additionally, the 14 base pair insertion/deletion polymorphism (14 bp I/D) emerged as a prognostic marker, with D/D genotype carriers showing lower survival rates compared to I/D and I/I genotype carriers. Our study highlights the clinical relevance of HLA-G polymorphisms in gastric cancer, suggesting their potential as prognostic markers and therapeutic targets. Further elucidation of HLA-G-related pathways could lead to personalized treatment strategies and improved patient outcomes in gastric cancer.

**Electronic supplementary material:**

The online version of this article (10.1007/s00262-024-03771-w) contains supplementary material, which is available to authorized users.

## Introduction

Gastric cancer ranks as the fifth most prevalent cancer worldwide, exhibiting an annual incidence of 11.1 new cases per 100,000 individuals. Furthermore, it stands as the 5th most lethal cancer, with 7.7 deaths per 100,000 individuals annually, underscoring its aggressive nature. The 5-year survival rate for this cancer subtype remains at 29.5%, persisting over the past three decades [1, 2].

HLA-G, a non-classical class I HLA gene, encodes molecules with tolerogenic properties. The HLA-G gene comprises 7 introns and 8 exons, and exon 8 constitutes an untranslated region (3'UTR) with regulatory properties [3]. Specific polymorphic sites within the 3'UTR region, such as the 14 base pair (14 bp) INS/DEL (I/D), + 3142C/G, and + 3187A/G polymorphisms, influence mRNA stability and HLA-G expression levels. These polymorphisms can give rise to extended haplotypes (denoted as UTR-1 to UTR-7), which either upregulate (UTR-1 or UTR-6) or downregulate (UTR-2 or UTR-5) HLA-G expression [4].

Previous research has documented HLA-G expression in gastric tumors [5]. Thus, investigating polymorphisms associated with expression levels, such as those within the 3’UTR of this molecule, could help identify new genetic markers implicated in the risk, severity, or prognosis of this pathology.

In this work, we focused on studying the influence of the aforementioned HLA-G UTR polymorphisms in paired tissue samples (tumoral and non-tumoral) obtained from a cohort of patients with gastric adenocarcinoma. Such an approach will allow us to assess the suitability of HLA-G variants as potential risk markers for gastric adenocarcinoma and elucidate their significance in the prognosis of this type of cancer.

## Patients, materials and methods

The study was approved by the local ethics committee and carried out in compliance with the ethical guidelines outlined in the Declarations of Helsinki. Prior to the collection of samples, all patients provided written informed consent.

A total of 111 patients with gastric cancer were included (see Table 1), alongside a control group of 119 healthy Spanish individuals. Inclusion criteria were: patients over 18 years old with gastric adenocarcinoma, confirmed by gastroscopy and preoperative biopsy, stratified according to UICC/AJCC criteria (7th edition 2009). In any TNM stage and Karnofsky index > 70% or performance status ≤ 2; while exclusion criteria included: non-resectability of the primary tumor, coexistence with other neoplastic diseases, pregnancy and severe alterations of hepatic, cardiovascular, or renal function. Control group individuals were selected among sex- and age-matched healthy donors, without any oncological or immune system-related pathology, all of them from the same geographic region (Spain).

**Table 1:** Demographic and clinical characteristics of patients

	Patient characteristics		
		Median	IQR (Min–Max)
Age (*y*)	Median (Min–Max)	70	61–77 (33–89)
		*N*	%
Sex, *N*. (%)	Male	66	(60.6%)
	Female	43	(55.4%)
TNM staging, *N*. (%)	I	24	(22.0%)
	II	39	(36.0%)
	III	23	(21.0%)
	IV	23	(21.0%)
Treatment a, *N*. (%)	Surgery	111	(100%)
	Chemotherapy	111	(100%)
Localization, *N*. (%)	Fundus	14	(13.6%)
	Antrum	37	(35.9%)
	Body	34	(33.0%)
	Diffuse	6	(5.8%)
	Cardia	4	(3.9%)
	Gastric Bypass	7	(6.8%)
Type, *N*. (%)	Intestinal	51	(52.7%)
	Non‐intestinal	52	(47.3%)
Overall survival, *N*. (%)	< 5 y	60	(58.3%)
	> 5 y	43	(41.7%)

Genomic DNA was extracted from paired (tumor and distal) tissues as well as blood samples obtained from patients with gastric cancer using the Nucleon BACC kit (GE Healthcare). DNA extraction from saliva samples collected from controls was performed using the Oragene DNA 500 kit (DNA Genotek) and purified with PrepIT-L2P (DNA Genotek). Quantity and purity of DNA was assessed spectrophotometrically in a Nanodrop-One (Thermo-Scientific).

HLA-G polymorphism analysis was performed using PCR (see Supplementary Table 1). The 14 bp polymorphism was analyzed by band size discrimination [6], and the remaining UTR SNPs were analyzed by Sanger sequencing. Primers and PCR conditions are detailed in Supplementary Table 1.

The PCR or PCR–RFLP data of the studied polymorphisms were analyzed using the software SNPStats. This software allows the evaluation of Hardy–Weinberg equilibrium (exact test), Chi-square test, odds ratio (OR) estimation for the association between polymorphisms, the analysis of linkage disequilibrium using the D statistic and correlation coefficient, and haplotype analysis employing the EM algorithm [7, 8].

Kaplan–Meier method was used to estimate the 5-year survival function of patients with gastric cancer considering various genetic factors (GraphPad Prism 8.0 software). Multivariate Cox regression models were used to simultaneously assess the effect of genetic factors along with other variables such as comorbidities, clinical features, and demographic characteristics on 5-year survival of patients (software R). In all Cox regression fits, both individual and global Schoenfeld test indicated that no covariate in the model nor the model as a whole violate the proportional hazard assumption, meaning that the hazard ratio stays constant over time [9].

*P*-values below 0.05 were considered statistically significant. When required, the Holm–Bonferroni (HB) sequential correction method for multiple testing was applied to the statistical analyses. This method compares the *k*-ranked *p*-value to the nominal significance level (0.05) divided by (*n* − *k* + 1).

## Results

### Hardy–Weinberg equilibrium and linkage disequilibrium

**Fig. 1:**
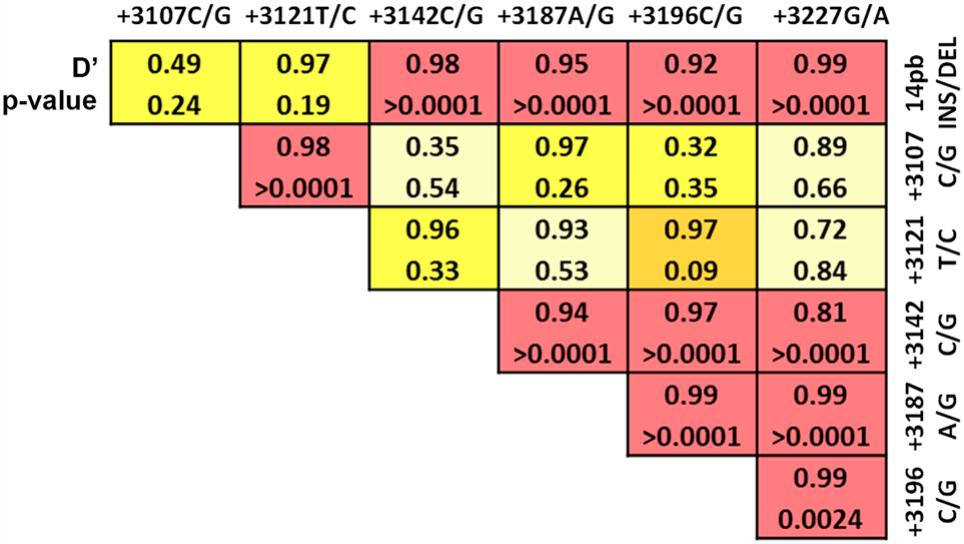
3’UTR SNPs are in linkage disequilibrium, forming a combined haplotype. According to the values obtained with the SNPStats software (D’ and r), these polymorphisms lie in close vicinity and are in linkage disequilibrium, allowing them to form a combined haplotype in our patients, as has been already described previously

The analysis showed that UTR polymorphisms were in Hardy–Weinberg equilibrium (not shown). Additionally, genetic distance analysis demonstrated that the polymorphisms were in linkage disequilibrium (Fig. 1), suggesting that these variants form combined haplotypes, as described in previous works. We then proceeded to analyze the distribution of these haplotypes in our study population.

### UTR-1 and 6 haplotypes are increased in patients with gastric cancer

The analysis of the 9 polymorphisms forming the HLA-G 3’UTR haplotypes showed an elevated frequency of UTR-1 and UTR-6, which are associated with increased HLA-G expression. Specifically, UTR-1 showed an odds ratio (OR) of 2.42 (1.12–5.21 95% CI, *p*-value = 0.025) and UTR-6 an OR of 3.02 (1.03–8.90 95% CI, *p*-value = 0.046), with frequencies of 32.1% and 6.9%, respectively, compared to the control group (25.5% and 4.6%, respectively) (Supplementary Table 2).

These data provide evidence of the role of the 3’UTR region polymorphisms of the HLA-G gene in gastric cancer susceptibility.

### Patients with the 14 bp D/D polymorphism show lower survival rate

Survival curve analysis showed that the 14 bp polymorphism was the only one implicated in the survival of patients with gastric cancer.

**Fig. 2:**
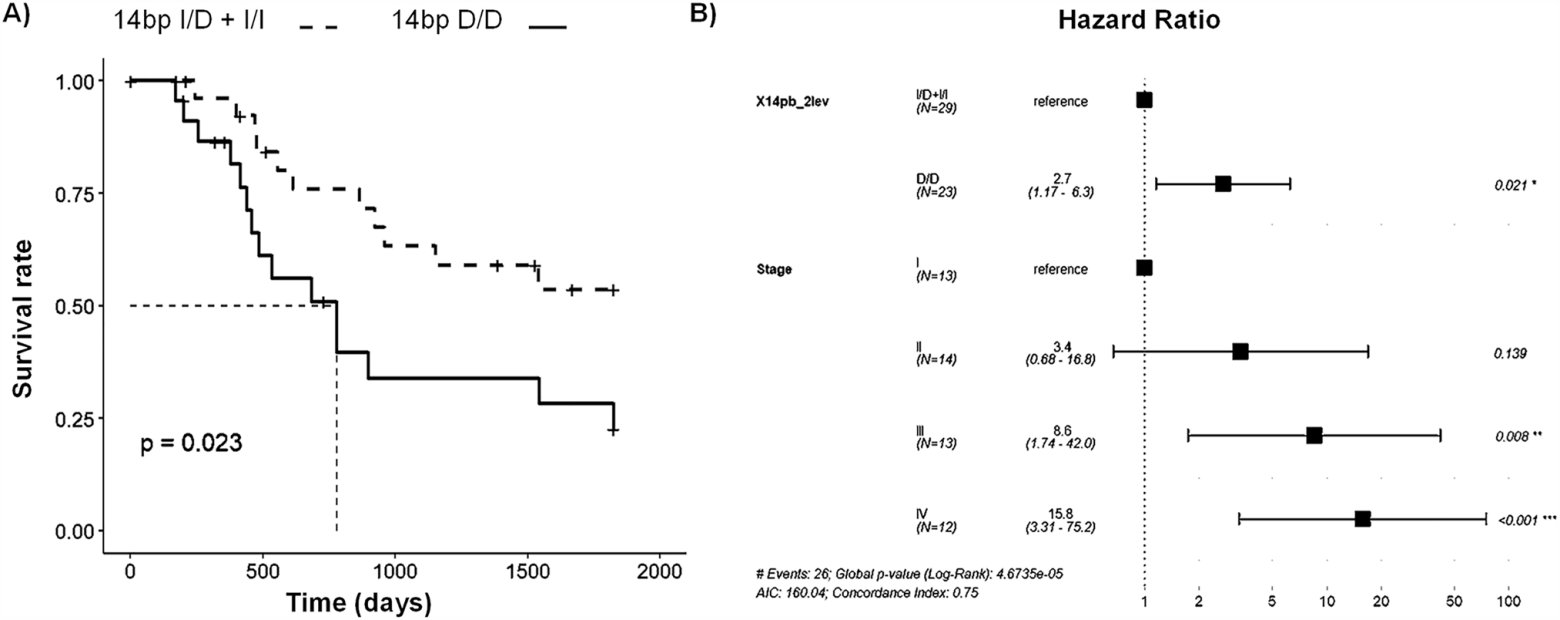
14 bp D/D genotype predicts a lower survival rate in patients with gastric cancer. **a** Kaplan–Meier curve depicting survival rates for 14 bp D/D and 14 bp I/D + I/I combined genotypes, showing a lower survival rate in patients with D homozygosity. **b** Cox regression analysis with 14 bp and TNM staging show an increased hazard ratio in patients with the 14 bp genotype compared to patients with the I/D or I/I genotype, with TNM as a cofactor implicated in survival

Specifically, patients with the 14 bp D/D genotype exhibited a lower survival rate (*p* = 0.023, survival below 25%), compared to 14 bp I/D and 14 bp I/I patients combined (survival above 50%) (Fig. 2a).

Moreover, the corresponding Cox regression model along with TNM staging showed that the hazard ratio estimate of D/D versus I/D + I/I is 2.7 (95% CI 1.17–6.3, *p*-value = 0.021) (Fig. 2b). Other demographic variables did not reach significance to be included in this model, meaning that only TNM and 14 bp I/D polymorphism were implicated in the risk of survival.

## Discussion

In recent years, significant interest has been directed toward the interplay between HLA-G, immunoediting, and cancer [10–12]. Indeed, the HLA-G-mediated signaling pathway is currently acknowledged as a novel therapeutic immune checkpoint, alongside other firmly established checkpoints [13].

This study shows that individuals harboring genetic markers in the 3’UTR region associated with elevated HLA-G expression are more susceptible to developing gastric cancer. In our cohort, we observed a heightened frequency of 3’UTRs inducing high HLA-G high expression (namely, UTR-1 and UTR-6,) among patients with gastric cancer,

The UTR-1 and 6 haplotypes displayed an increased frequency in patients with gastric cancer. These haplotypes encompass variants known to increase HLA-G levels [4], thereby fostering an HLA-G-mediated immunosuppressive microenvironment, as proposed in our initial hypothesis.

Several authors have identified mechanisms that regulate HLA-G expression probably related to the 3’UTR variants. For instance, the 14 bp I/D is associated with mRNA stability [14], while + 3142 G/C and + 3187 G/A (located in an AU-rich element, ARE) may participate in miRNA-mediated posttranscriptional regulation [15] or in mRNA degradation [16], respectively. Additionally, other SNPs (namely, + 3001 C/T, + 3003 T/C, + 3010 G/C, + 3027 C/A, + 3035 C/T, and + 3196 C/G), have been proposed as miRNA targets, suggesting a potential role in posttranscriptional regulation of HLA-G [17].

Elevated HLA-G levels have been linked to numerous physiological [18] and pathological conditions, such as cancer, while lower levels are associated with autoimmune and inflammatory diseases [19], in line with the results described herein.

These findings underscore HLA-G as a promising target for potential therapeutic interventions. Strategies such as miRNA-mediated downregulation of HLA-G expression or inhibition of its interaction with its cognate receptors, akin to current PD-1/PD-L1 immunotherapies, hold promise for stimulating immune responses against the tumor.

Furthermore, exploring the functional implications of these HLA-G polymorphisms and their relationship with disease susceptibility and progression could shed light on the association between HLA-G markers and pathological conditions.

Other works and meta-analysis described an association of HLA-G 3’UTR haplotypes to the development and severity of different malignancies, such as breast and colorectal cancer, were 14 bp Del/Del and + 3142 C/C, both forming UTR-1 and UTR6 haplotypes, showed association to risk of developing both cancers [20, 21]. Moreover, other authors showed an association of HLA-G levels of expression and the 14 bp polymorphism in gynecologic cancers [22], and, in the same vein of thinking, loss of miR-152 increases HLA-G expression in vitro [23], mimicking the effect of + 3142 polymorphisms, as miR-152 is not able to bind + 3142 C/C.

All these findings not only support the results of our work, but also provide a functional explanation for them, as it seems clear that there is a link between variants in the 3'UTR of HLA-G and the expression levels of this protein and, consequently, with the risk of developing tumors of epithelial origin.

This work has some limitations. First, it is focused on genetic variants with a known effect on HLA-G expression. However, further studies in a large cohort of patients may enable to link this polymorphisms and UTR haplotypes with data of protein in sera and tissue of patients, thus comparing the actual effect of these variants in the level of expression of HLA-G. Although it is an interesting point, several factors can modify HLA-G levels of expression that diverge from the regulation of classic class I HLA molecules [24], such as inflammation and cellular stress [23, 25], making it difficult to link the effect of genetic variants with HLA-G expression in the context of an evolving pathology like cancer.

Moreover, HLA-G gene is in a high linkage disequilibrium with other class I genes, as all of them are located in the same chromosome. For instance, the HLA-A locus is surrounded by HLA class Ib genes: HLA-E, HLA-H, HLA-G, and HLA-F [26], meaning that the association of genetic variants to a certain phenotype may be mediated by other genes in that haplotype.

Finally, although several authors described the effect of HLA-G in the immune system [27, 28], functional analysis may enlight the actual immunomodulatory properties of this molecule in the context of gastric cancer. In our group, we already described the expression of HLA-G in gastric tumors (6, Supplementary Material), and a closer look to the in vivo effect of the immunomodulatory properties of HLA-G in the tumor microenvironment should address the importance of this molecule in the evolution and possible therapies of this pathology.

## Electronic supplementary material

Below is the link to the electronic supplementary material.

Supplementary file 1 (DOCX 13 kb)

Supplementary file 2 (DOCX 16 kb)

## Data Availability

Data is provided within the manuscript or supplementary information files. Any additional data is available upon request from the authors.
