# Supplementary material for: HLA-G high-expressor 3’UTR markers are linked to gastric cancer development and survival
Source: Cancer Immunol Immunother. 2024 Nov 16;74(1):26. doi: 10.1007/s00262-024-03771-w (PMC11569108; doi:10.1007/s00262-024-03771-w)
Supplement: Supplementary file 1 — (DOCX 13 kb) [file 262_2024_3771_MOESM1_ESM.docx]

Table 1. Primers and PCR programs employed in this study.

| **Polymorphism** | **Primers** | | **PCR conditions** | | | | |
| --- | --- | --- | --- | --- | --- | --- | --- |
|  | **Forward** | **Reverse** | **Denaturalization** | | **Annealing** | **Elongation** | |
| **14bp INS/DEL (30 cycles)** | 5’-GTGATGGGCTGTTTAAAGTGTCACC -3’ | 5’-GGAAGGA ATGCAGTTC AGCATGA -3’ | 94°C | | 64°C | 72°C | |
|  |  |  | 2 min | 30 sec | 60 sec | 60 sec | 10 min |
| **UTR SNPs**  **(32 cycles)** | 5’-CATGCTG AACTGCAT TCCTTCC -3’ | 5’-CTGGTGG GACAAGGT TCTACTG -3’ | 94°C | | 65.5°C | 72°C | |
|  |  |  | 5 min | 30 sec | 30 sec | 60 sec | 5 min |
