# Supplementary material for: HLA-G high-expressor 3’UTR markers are linked to gastric cancer development and survival
Source: Cancer Immunol Immunother. 2024 Nov 16;74(1):26. doi: 10.1007/s00262-024-03771-w (PMC11569108; doi:10.1007/s00262-024-03771-w)
Supplement: Supplementary file 2 — (DOCX 16 kb) [file 262_2024_3771_MOESM2_ESM.docx]

Table 2. HLA-G 3’-UTR haplotype frequencies estimation and distribution. UTR 1 and 6 are overrepresented in patients with gastric cáncer.

| **3'UTR Haplotypes analysis** | | | | | | | | | | | | | | |
| --- | --- | --- | --- | --- | --- | --- | --- | --- | --- | --- | --- | --- | --- | --- |
| **UTR** | **14bp** | **+3092 G>T** | **+3107 C>G** | **+3111 G>A** | **+3121 T>C** | **+3142 C>G** | **+3187 A>G** | **+3196 C>G** | **+3227 G>A** | **Control** | **Gastric Cancer** | **Mean Freq.** | **OR (95% CI)** | **P-value** |
| 5 | I | G | C | G | T | G | A | C | G | 0.116 | 0.069 | 0.094 | 1.00 | --- |
| **1** | **D** | **G** | **C** | **G** | **T** | **C** | **G** | **C** | **G** | 0.254 | 0.321 | 0.284 | **2.42 (1.12-5.21)** | **0.025** |
| 2 | I | G | C | G | T | G | A | G | G | 0.254 | 0.235 | 0.245 | 1.82 (0.84-3.93) | 0.131 |
| 3 | D | G | C | G | T | G | A | C | G | 0.149 | 0.135 | 0.142 | 1.75 (0.75-4.09) | 0.195 |
| 4 | D | G | C | G | T | C | A | C | G | 0.126 | 0.131 | 0.128 | 2.11 (0.85-5.24) | 0.107 |
| **6** | **D** | **G** | **C** | **G** | **T** | **C** | **A** | **C** | **A** | 0.046 | 0.069 | 0.060 | **3.02 (1.03-8.90)** | **0.046** |
